# Wistar rats: a forgotten model of age-related hearing loss

**DOI:** 10.3389/fnagi.2014.00029

**Published:** 2014-03-05

**Authors:** Juan C. Alvarado, Verónica Fuentes-Santamaría, María C. Gabaldón-Ull, José L. Blanco, José M. Juiz

**Affiliations:** Facultad de Medicina, Instituto de Investigación en Discapacidades Neurológicas, Universidad de Castilla-La ManchaAlbacete, Spain

**Keywords:** rat model, auditory brainstem response (ABR), vesicular transport proteins, presbyacusis, hearing loss, cochlear nucleus

## Abstract

Age-related hearing loss (ARHL) is one of the most frequent sensory impairments in senescence and is a source of important socio-economic consequences. Understanding the pathological responses that occur in the central auditory pathway of patients who suffer from this disability is vital to improve its diagnosis and treatment. Therefore, the goal of this study was to characterize age-related modifications in auditory brainstem responses (ABR) and to determine whether these functional responses might be accompanied by an imbalance between excitation and inhibition in the cochlear nucleus of Wistar rats. To do so, ABR recordings at different frequencies and immunohistochemistry for the vesicular glutamate transporter 1 (VGLUT1) and the vesicular GABA transporter (VGAT) in the ventral cochlear nucleus (VCN) were performed in young, middle-aged and old male Wistar rats. The results demonstrate that there was a significant increase in the auditory thresholds, a significant decrease in the amplitudes and an increase in the latencies of the ABR waves as the age of the rat increased. Additionally, there were decreases in VGLUT1 and VGAT immunostaining in the VCN of older rats compared to younger rats. Therefore, the observed age-related decline in the magnitude of auditory evoked responses might be due in part to a reduction in markers of excitatory function; meanwhile, the concomitant reduction in both excitatory and inhibitory markers might reflect a common central alteration in animal models of ARLH. Together, these findings highlight the suitability of the Wistar rat as an excellent model to study ARHL.

## Introduction

Age-related hearing loss (ARHL), or presbyacusis, is a hearing condition characterized by a progressive increase in auditory thresholds that occurs during aging (for a review, see Boettcher, 2002; Syka, 2002; Gordon-Salant, 2005; Bielefeld et al., 2010; Huang and Tang, 2010; Sprinzl and Riechelmann, 2010; Fetoni et al., 2011). According to the WHO (2013), presbyacusis is one of the most frequent sensory impairments in elderly people, with an estimate of about one-third of people over 65 years old suffering from some degree of this disability. ARHL is a source of important socio-economic consequences, leading to a decrease in quality of life and presenting an important economic burden on public health care (WHO, 2002, 2013; Huang and Tang, 2010). Understanding the mechanisms that occur in the central auditory pathway associated to aging is essential to improve the diagnosis and treatment of patients with ARHL.

One approach to study ARHL is the use of animal models that, similar to humans, suffer from a progressive decline in hearing, reflected as an increase in auditory thresholds, as age increases (for review see Syka, 2002, 2010; Ohlemiller, 2006; Bielefeld et al., 2010; Fetoni et al., 2011). Although not all data obtained from animal models can be generalized to humans, animal models are one of the best experimental tools for the evaluation of ARHL. In support of this idea, valuable information regarding the physiological, histological, genetic, and molecular mechanisms involved in presbyacusis have been obtained from studies performed in rodents, including guinea pigs, chinchillas, Mongolian gerbils, mice, and rats (Syka, 2002, 2010; Ohlemiller, 2006; Bielefeld et al., 2008, 2010; Fetoni et al., 2011). Nevertheless, most of the findings regarding this sensory impairment have been derived from studies carried out in mice and rats.

Several strains of rats are commonly used for the evaluation of auditory pathologies. However, only the inbred albino rat strain Fischer 344 (F344) has gained acceptance as a useful animal model for the study of auditory modifications related to aging (for review, see Syka, 2002; Bielefeld et al., 2010; Fetoni et al., 2011). With a lifespan of 22–28 months, the fast and progressive hearing impairment in the F344 rat begins in the high frequencies at approximately 12 months of age and then progresses to the low frequencies between 12 and 18 months. By 24 months of age, brainstem responses (ABR) show a threshold shift of up to 60 dB in the high frequencies and up to 40 dB in the low frequencies. Additionally, pronounced age-related changes in other ABR parameters, such as wave amplitude and latencies, have been described in F344 rats. Studies in the central auditory system of this strain, specifically in the cochlear nuclei (CN), have demonstrated age-related alterations in inhibitory and excitatory neurotransmission, including a dysfunctional glycine receptor that might be the result of modifications in the expression of its subunits, a reduction of approximately 29% of glycine with a concomitant reduction of approximately 24% of glutamate in aged F344 rats compared to younger F344 rats, and no apparent loss of synapses or dendrites in older animals (Krenning et al., 1998; Helfert et al., 2003; Bielefeld et al., 2010; Syka, 2010; Fetoni et al., 2011). Although less frequently, the outbred albino rat strain Sprague-Dawley has also been used to evaluate presbyacusis. The few studies available have shown that the Sprague-Dawley strain exhibits a loss of hair cells and spiral ganglion neurons that increases with age (Keithley and Feldman, 1982; Fetoni et al., 2011). Regarding the outbred pigmented rat strain Long Evans, the contribution of this strain to the study of the ARHL is minimal, as these animals exhibit worse auditory acuity than other strains and display very limited hearing impairment with age (Overbeck and Church, 1992; Buckiova et al., 2007; Fetoni et al., 2011).

Surprisingly, despite the fact that the outbred albino rat strain Wistar is also a widely used experimental model in the auditory system (Borg, 1982; Chen and Chen, 1991; Newton et al., 1992; Overbeck and Church, 1992; Pukkila et al., 1997; Fujioka et al., 2006; Kujawa and Liberman, 2006; Church et al., 2007, 2010, 2012a,b; Hougaard et al., 2007; Bielefeld et al., 2008; Hu and Cai, 2010), there are no studies available regarding its use as an animal model for the study of ARHL. Therefore, the goal of this study was to investigate age-related physiological and histological changes in this strain. Specifically, we aimed to determine whether the Wistar strain could be a reliable model contributing to the understanding of the mechanisms involved in the initiation and development of ARHL. Toward this goal, we characterized in detail, possible functional alterations in auditory thresholds, wave amplitudes and latencies using ABR recordings, and we also evaluated the concomitant histological modifications in excitatory and inhibitory synaptic vesicular transporters in the CN.

## Methods

### Animals

Data were obtained from 24 adult male Wistar rats purchased from Charles River (Barcelona, Spain) and housed at the University of Castilla-La Mancha (Albacete, Spain) on a 12/12 h light/dark cycle with free access to food and water. According to age, the rats were distributed into three groups: 6- to 8-month-old (*n* = 8), 12- to 14-month-old (*n* = 8) and 18- to 20-month-old (*n* = 8). All procedures used were approved by the Ethics Committee on Animal Experimentation of the University of Castilla-La Mancha (Permit Number: PR-2013-02-03) and conformed to Spanish (R.D. 53/2013; Law 32/2007) and European Union (Directive 2010/63/EU) regulations for the care and use of animals in research.

### Physiological procedures

#### Auditory brainstem response (ABR) recordings

ABR recordings were performed as described previously (Alvarado et al., 2012). Briefly, a sound-attenuating, electrically shielded booth (EYMASA/INCOTRON S.L., Barcelona, Spain) placed inside a sound-attenuating room was used. Anesthesia was induced (4%) and maintained (1.5–2%) with isoflurane (1 L/min O_2_ flow rate). Subdermal needle electrodes (Rochester Electro-Medical, Tampa, FL, USA) were placed at the vertex (non-inverting) and in the right (inverting) and the left (ground) mastoids. Acoustic stimulation and recordings were performed using a BioSig System III (Tucker-Davis Technologies, Alachua, FL, USA). Acoustic stimuli consisted of tone bursts (5 ms rise/fall time without a plateau with a cos2 envelope delivered at 20/s) of different frequencies (0.5, 1, 2, 4, 8, 16, and 32 kHz) that were generated digitally using SigGenRP software (Tucker-Davis Technologies) and RX6 Piranha Multifunction Processor hardware (Tucker-Davis Technologies). Stimuli were delivered into the external auditory meatus of the right ear using an EDC1 electrostatic speaker driver (Tucker-Davis Technologies) through an EC-1 electrostatic speaker (Tucker-Davis Technologies). Prior to the experiments, stimuli were calibrated using SigCal software (Tucker-Davis Technologies) and an ER−10B+ low noise microphone system (Etymotic Research Inc., Elk, Groove, IL, USA). The evoked potentials were filtered (0.3–3.0 kHz), averaged (500 waveforms) and stored for offline analysis. During the recording, the temperature was monitored using a rectal probe and maintained at 37.5 ± 1°C using a non-electrical heating pad.

#### ABR thresholds, amplitudes, and latencies

Auditory thresholds, as well as amplitudes and latencies for all waves that comprise the ABR, were calculated for each of the frequencies evaluated. To determine *the auditory threshold level*, the background activity (measured before the stimulus onset) and the evoked responses were recorded in 5 dB steps descending from 80 dB sound pressure level (SPL). The auditory threshold was defined as the stimulus intensity that evoked waveforms with a peak-to-peak voltage greater than 2 standard deviations (SD) of the background activity (Cediel et al., 2006; Garcia-Pino et al., 2010; Alvarado et al., 2012). *The wave amplitude* was defined as the peak-to-peak amplitude from the positive peak to the subsequent negative trough of each wave (Popelar et al., 2008; Church et al., 2010; Alvarado et al., 2012). *Two latencies* were measured for each ABR wave: (1) the latency comprising the time between the stimulus onset and the positive peak, and (2) the latency comprising the time between the stimulus onset and the negative trough (Chiappa et al., 1979; Chen and Chen, 1991; Gourévitch et al., 2009; Alvarado et al., 2012). In addition, using the positive and negative individual latencies of each wave, *the interpeak latencies* between I-II, II-IV, and I-IV waves were calculated. An acoustic transit time of 0.5 ms between the speaker's diaphragm and the rat's tympanic membrane was added to the latencies.

### Histological procedures

#### Characterization of antibodies

The primary antibody sources, host species and dilutions used in the present study are shown in Table 1. Presynaptic and post-synaptic antibodies included (1) rabbit anti-calretinin (CR); (2) goat anti-CR; (3) guinea pig anti-vesicular glutamate transporter 1 (VGLUT1) and (4) rabbit anti-vesicular GABA transporter (VGAT).

**Table 1:** **List of primary antibodies**.

**Primary antibody**	**Immunogen**	**Host**	**Code/clone**	**Dilution**	**Manufacturer**
CR	Recombinant human CR	Rabbit	7699/3H	1:1500	Swant, Bellinzona, Switzerland
CR	Rat CR	Goat	AB1550	1:2000	Millipore, Billerica, MA, USA
VGLUT1	Amino acid residues 541–560 of rat vGLUT1	Guinea pig	AB5905	1:1500	Millipore, Billerica, MA, USA
VGAT	Rat VGAT	Rabbit	AB5062P	1:1000	Millipore, Billerica, MA, USA

The polyclonal anti-CR antibodies 7699/3H and AB1550 were raised against the CR protein from humans and rats, respectively. These antibodies recognize a single 31-kDa band that corresponds to the CR protein via western blot analysis of membrane fractions from the cochlear nucleus (Schwaller et al., 1994; Fuentes-Santamaría et al., 2005a). CR is a calcium-binding protein that is distributed in somata and neurites of auditory neurons in various species, including mammals (Baimbridge et al., 1992; Winsky and Jacobowitz, 1995; Lohman and Friauf, 1996; Henkel and Brunso-Bechtold, 1998), and appears to play a role in the control of calcium homeostasis by buffering the calcium that enters cells during synaptic activation (Baimbridge et al., 1992; Miller, 1995). The staining pattern of this antibody matched previous descriptions of CR immunostaining in the CN (Fuentes-Santamaría et al., 2005a).

The guinea pig anti-VGLUT1 (AB5905) antibody was raised against a synthetic peptide of the C-terminal domain of the rat VGLUT1 protein. In western blots of a synaptic membrane fraction from the rat cerebral cortex, VGLUT1, which is a well-known marker of excitatory synapses (Kaneko and Fujiyama, 2002; Kaneko et al., 2002), recognizes a single band of 60 kDa molecular weight (Melone et al., 2005). The staining pattern described herein is in agreement with previous reports in the CN (Zhou et al., 2007) and other auditory nuclei (Altschuler et al., 2008; Hackett et al., 2011; Ito et al., 2011).

The rabbit anti-VGAT (AB5062P) antibody was raised against a 17 amino acid peptide sequence near the C-terminal region of rat VGAT (McIntire et al., 1997). This antibody recognizes a single 57 kDa band via western blot of rat retina lysates (McIntire et al., 1997; Chaudhry et al., 1998). This antibody has been extensively used to label both GABAergic and glycinergic synapses (Wang et al., 2009; Ito et al., 2011).

#### Immunohistochemistry

Rats were anesthetized via an intraperitoneal injection of ketamine (100 mg/Kg) and xylazine (5 mg/Kg) and transcardially perfused with 0.9% saline followed by a fixative solution of 4% paraformaldehyde in 0.1 M phosphate buffer (PB, pH 7.3). The brains were removed and cryoprotected overnight with 30% sucrose. Coronal sections (40 μm thick) were generated using a sliding microtome and processed in four alternating series; the first two series of sections were processed for either VGLUT1 or VGAT immunostaining, and the other two series were used for double-labeling studies. After blocking for 1 h in a solution containing 10% normal horse serum diluted in Tris-buffered saline (TBS, pH 7.4) with 0.2% Triton X-100 (0.2% TBS-Tx), the sections were subsequently incubated overnight at 4°C in the same buffer solution with either the VGLUT1 (1:1500) or VGAT (1:1000) antibody. The next day, the sections were washed in 0.2% TBS-Tx solution and incubated for 2 h at room temperature in the anti-guinea pig or anti-rabbit secondary antibody, respectively (1:200; Vector Laboratories, Burlingame, CA, USA). Then, after several rinses in 0.2% TBS-Tx, the sections were incubated in an avidin-biotin complex for 1 h, and the immunoreaction was visualized using diaminobenzidine (DAB). The exposure time to DAB was similar across samples. Finally, the sections were washed thoroughly, mounted on gelatin-coated slides, air-dried, dehydrated using ethanol, cleared using xylene and coverslipped using Cytoseal® (Stephens Scientific, Wayne, NJ, USA). Three sets of control experiments were performed to test the specificity of the immunohistochemical detection system: (1) omission of the primary antibody via replacement with TBS-BSA; (2) omission of secondary antibodies; and (3) omission of ABC reagent. No immunostaining was detected under these conditions.

#### Double-labeling

Sections were rinsed several times in 0.2% TBS-Tx and blocked for 1 h in the same buffer solution containing 10% normal goat serum. Then, the sections were incubated overnight in a solution of primary antibodies against CR and either VGLUT-1 or VGAT. Following four 15 min rinses in 0.2% TBS-Tx, the sections were incubated in a cocktail of fluorescently labeled secondary antibodies for 2 h at room temperature (1:200, anti-rabbit conjugated to Alexa 488 for CR and anti-guinea pig conjugated to Alexa 594 for VGLUT1, or anti-goat conjugated to Alexa 488 for CR and anti-rabbit conjugated to Alexa 594 for VGAT; Molecular Probes, Eugene, OR, USA). After several rinses in TBS, the sections were mounted, coverslipped and stored overnight at 4°C. Immunofluorescence sections were examined under a Zeiss LSM 710 laser scanning confocal microscope (Zeiss, Germany) and images were analyzed using the ZEN 2009 Light Edition software (Zeiss, Germany).

## Analysis of immunostaining

###  

#### Imaging

Immunostained sections were examined via brightfield illumination using a Nikon Eclipse 80*i* photomicroscope (Nikon Instruments Europe B.V.) with a 40× objective, and images were captured using a DXM 1200C1200C digital camera (Nikon Instruments Europe B.V.) that was attached to the microscope. Color images of each field were digitized, and the resultant 8-bit image from the red channel, containing a grayscale of pixel intensities from 0 (white) to 255 (black), was used for densitometric analysis.

#### Densitometric analysis

The densitometry procedure to evaluate immunostaining was performed as described elsewhere (Fuentes-Santamaría et al., 2003, 2005a,b, 2007a,b, 2008; Alvarado et al., 2004, 2005, 2007, 2009) using the public domain image analysis software Scion Image for Windows (version beta 4.0.2; developed by Scion Corp). The CN subdivisions were defined based on previous studies (for review, see Cant and Benson, 2003). The analysis of VGLUT1 and VGAT immunostaining was performed on equally spaced coronal sections, 160 μm apart, extending throughout the rostrocaudal dimension of the anterior (AVCN) and posterior ventral cochlear nucleus (PVCN). For each section, three fields (dorsal, middle and ventral) were sampled using a 40× objective. To perform an appropriate comparison of VGLUT1 and VGAT immunostaining across samples, a macro was designed to process and analyze the captured images (Alvarado et al., 2004). First, the images were normalized. Then, the threshold level was set at two SD above the mean gray level of the field, and immunostained profiles exceeding this threshold were identified as labeled (Alvarado et al., 2004). For each field, two quantitative indexes were measured: (1) *the mean gray level of VGLUT1 and VGAT immunostaining*, which was used as an indirect indicator of protein levels within synaptic terminals, and (2) *the area of VGLUT1 and VGAT immunostaining*, calculated as the summed area of all profiles labeled above the threshold in each field, which provides an estimate of the area in which VGLUT1 and VGAT are expressed.

### Preparation of figures and statistical analysis

Photoshop (Adobe v5.5) and Canvas (Deneba v6.0) software were used to adjust the size, brightness and contrast of the images for the preparation of figures. All data are expressed as the means ± SD. The measurements of the amplitudes and latencies were performed at 80 dB SPL unless otherwise indicated. Comparisons between groups were performed using a one-way analysis of variance (ANOVA) with Scheffé *post-hoc* analysis as necessary. Statistical significance was defined as *p* < 0.05.

## Results

### Auditory thresholds

**Figure 1:**
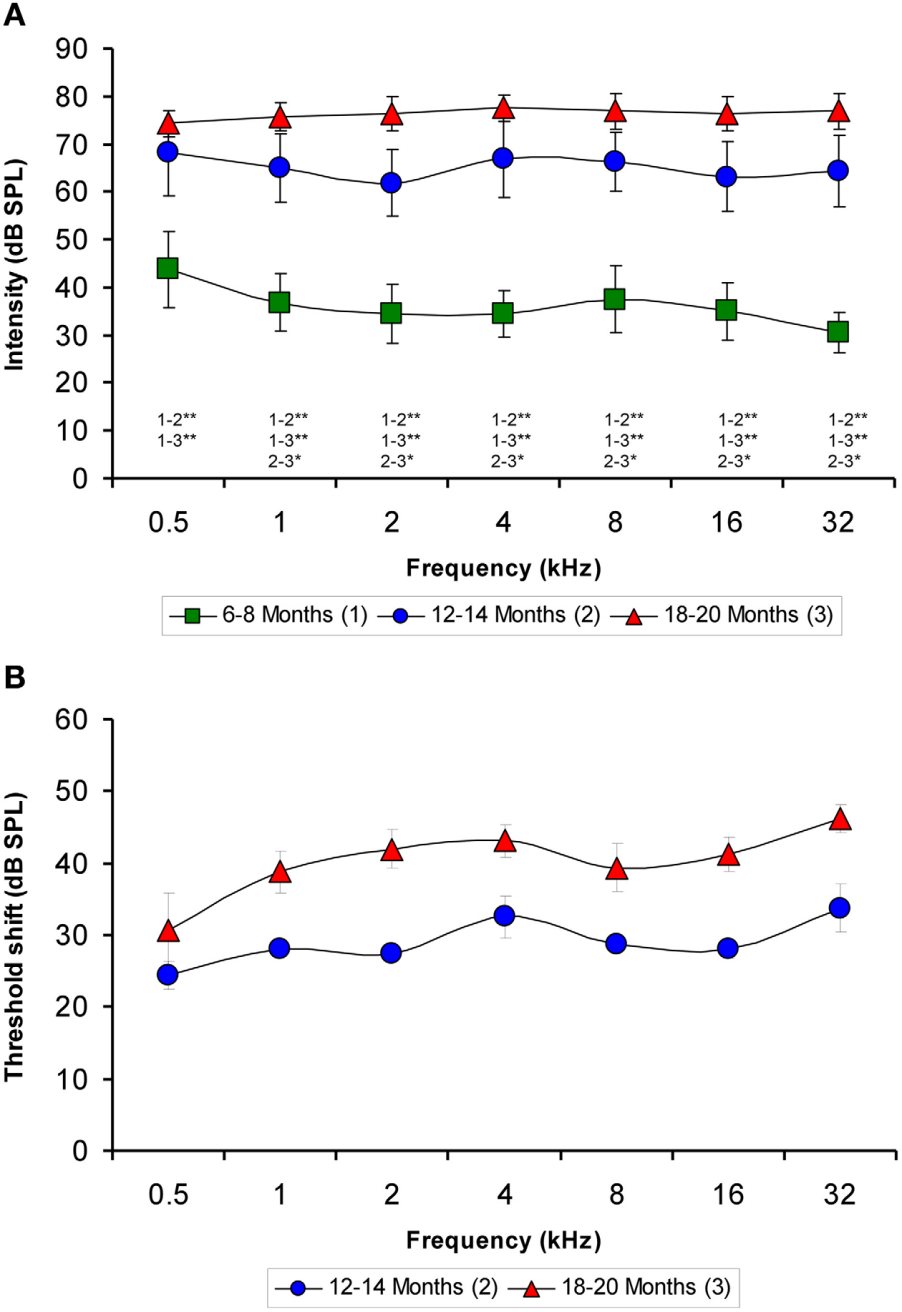
**Line graphs illustrating the relationship between the auditory thresholds and the frequencies tested for each age group**. Note that the mean values of the older groups rose as the age of the rats increased **(A)**. Compared to the 6- to 8-month-old rats, the threshold shift in the 12- to 14-month-old rats ranged from approximately 24–33 dB and in the 18- to 20-month-old rats rose from approximately 30–46 dB **(B)**. ^*^p < 0.05; ^**^p < 0.01.

Although the mean values of the auditory thresholds of 6- to 8-month-old rats were similar to those described previously for Wistar rats (Jamesdaniel et al., 2008; Church et al., 2010, 2012a; Alvarado et al., 2012; Pilati et al., 2012), the mean values of the auditory thresholds of the older groups increased as a function of age at all the frequencies evaluated (Figure 1A). When the mean auditory thresholds were plotted as a function of the stimulus frequency, the average thresholds of 6- to 8-month-old rats decreased from 43.75 ± 7.91 to 30.63 ± 4.17 dB as the frequency of the stimulus increased (Figure 1A). However, in both the 12- to 14-month-old and the 18- to 20-month-old groups of rats, the average thresholds were very similar across frequencies, ranging from 61.88 ± 6.89 to 68.13 ± 8.84 dB in the 12- to 14-month-old rats and from 74.38 ± 2.77 to 77.50 ± 2.67 dB in the 18- to 20-month-old rats (Figure 1A). The threshold shift in the older rats compared to the 6- to 8-month-old rats was from 24.38 ± 1.93 to 33.75 ± 3.40 dB for the 12- to 14-month-old group and from 30.63 ± 5.14 to 46.25 ± 1.95 dB for the 18 to 20-month-old group (Figure 1B). ANOVA analysis revealed a significant interaction between age and auditory threshold (Table 2). Accordingly, in the 18- to 20-month-old rats the mean auditory thresholds were significantly higher than those in the 6- to 8-month-old rats at all frequencies tested and were also significantly higher than the mean auditory thresholds in the 12- to 14-month-old rats at all frequencies except for 0.5 kHz. In addition, the mean auditory thresholds of the 12- to 14-month-old rats were also significantly higher than those of the 6- to 8-month-old rats at all frequencies tested (Figure 1A).

**Table 2:** **ANOVA analysis of the interaction between age of the rats and ABR auditory thresholds**.

	**Frequencies (kHz)**						
	**0.5**	**1**	**2**	**4**	**8**	**16**	**32**
*F*_(2, 21)_ =	43.71^***^	45.86^***^	32.28^***^	69.59^***^	34.27^***^	27.79^***^	44.92^***^

*** p < 0.001.

### Wave amplitudes

**Figure 2:**
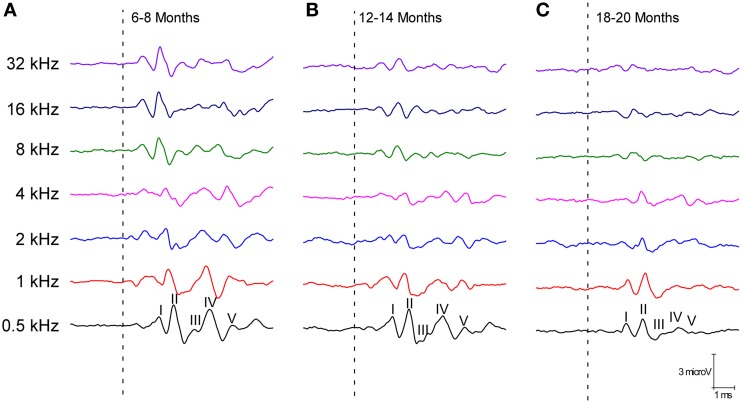
**Line graphs showing examples of ABR recordings of each age group at 80 dB SPL for all frequencies tested**. In the three experimental groups, the recordings displayed a distinctive pattern characterized by 4–5 evoked waveforms after the stimulus onset. Despite the similarities between the groups, there was an apparent decrease in the wave amplitudes with age. The ABR recordings were compared between the 6- to 8-month-old rats **(A)** and both the 12- to 14-month-old **(B)** and the 18- to 20-month-old rats **(C)**. Dashed lines indicate the stimulus onset. Stimulus intensity = 80 dB SPL.

**Figure 3:**
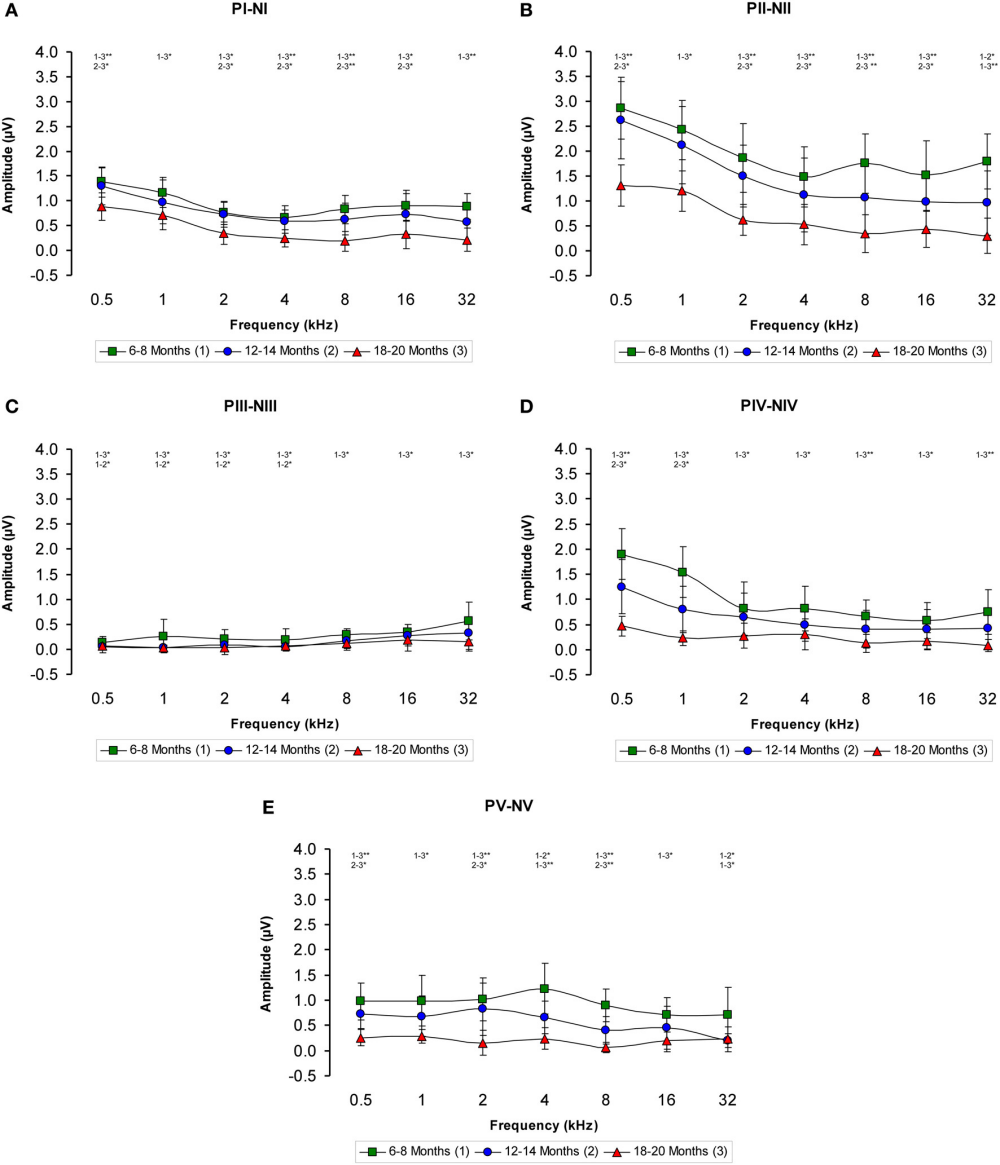
**Line graphs showing the wave amplitudes (μV) of each age group plotted as a function of frequency**. In the 6- to 8-month-old rats, the mean amplitudes of all waves were higher compared to the 18- to 20-month-old group at all frequencies studied and higher than in the 12- to 14-month-old rats for several of the frequencies evaluated **(A–E)**. Stimulus intensity = 80 dB SPL. ^*^p < 0.05; ^**^p < 0.01.

The ABR recordings of the three experimental groups evaluated displayed a distinctive pattern characterized by four to five evoked waveforms following the stimulus onset (Figure 2). Consistent with previous findings (Overbeck and Church, 1992; Church et al., 2010, 2012a,b, 2013; Alvarado et al., 2012), wave II was the largest of all waves, followed by waves I, IV, V, and finally by wave III, which was the smallest of the waves that comprised the ABR (Figure 2). However, despite the similarities among the three groups, there was an apparent decrease in the amplitude of all waves as the age of the rats increased (compare Figures 2A–C). To confirm this observation, a detailed evaluation was performed on the wave amplitudes of all frequencies studied in the three groups of rats. When mean wave amplitudes of each of the five frequencies analyzed was plotted as a function of the stimulus frequency, the mean amplitudes of the 6- to 8-month-old rats were higher compared to the 12- to 14-month-old and the 18- to 20-month-old rats (Figures 3A–E). Note that the wave amplitudes of the 18- to 20-month-old rats were the lowest of all (Figures 3A–E).

There was a statistically significant interaction between age and amplitude of the ABR wave. Specifically, for the wave I there were significant differences between the groups (Table 3), being the amplitudes of wave I in the 18- to 20-month-old group of rats smaller than those in the 6- to 8-month-old group of rats at all frequencies evaluated and smaller than those in the 12- to 14-month-old group of rats at 0.5, 2, 4, 8, and 16 kHz. No differences were detected in the wave I amplitudes between the 6- to 8-month-old and 12- to 14-month-old rats (Figure 3A). For the wave II amplitudes, there were also significant differences between the groups (Table 3), being the amplitudes of wave II in the 18- to 20-month-old group of rats smaller than those in the 6- to 8-month-old group of rats at all frequencies tested and smaller than those in the 12- to 14-month-old rats group at 0.5, 2, 4, 8, and 16 kHz. In the 12- to 14-month-old rats, the amplitude of wave II was smaller than that in the 6- to 8-month-old rats at 32 kHz (Figure 3B). For the wave III amplitudes, significant differences were observed between the groups (Table 3). At all the frequencies studied, smaller wave III amplitudes were detected in the 18- to 20-month-old rats compared to the 6- to 8-month-old rats. Additionally, the wave III amplitudes in the 12- to 14-month-old group of rats were smaller than those in the 6- to 8-month-old group of rats at 0.5, 1, 2, and 4 kHz. No differences were observed between the 12- to 14-month-old and the 18- to 20-month-old rats at any of the frequencies evaluated (Figure 3C). Regarding wave IV, ANOVA analysis also revealed significant differences between the groups (Table 3). The wave IV amplitudes in the 18- to 20-month-old rats were smaller than those in the 6- to 8-month-old rats at all frequencies evaluated and were smaller than the wave IV amplitudes in the 12- to 14-month-old rats at 0.5 and 1 kHz. No differences were detected between the 6- to 8-month-old and 12- to 14-month-old rats at any of the frequencies examined (Figure 3D). Finally, and similar to the other waves described above, significant differences were detected in the wave V amplitudes between the three groups (Table 3). Specifically, the amplitudes of wave V in the 18- to 20-month-old rats were smaller than the amplitudes of wave V in the 6- to 8-month-old rats at all frequencies examined and were smaller than those in the 12- to 14-month-old rats at 0.5, 2, and 8 kHz. The amplitudes of wave V in the 12- to 14-month-old rats were smaller than the amplitudes of wave V in the 6- to 8-month-old rats at 4 and 32 kHz (Figure 3E).

**Table 3:** **ANOVA analysis of the interaction between age of the rats and ABR wave amplitudes**.

		**Frequencies (kHz)**						
**Waves**		**0.5**	**1**	**2**	**4**	**8**	**16**	**32**
I	*F*_(2, 21)_ =	7.23^**^	4.32^*^	5.62^*^	6.55^**^	11.31^***^	4.43^*^	9.17^***^
II	*F*_(2, 21)_ =	6.52^**^	3.92^*^	4.82^*^	4.04^*^	9.18^**^	5.69^*^	12.13^***^
III	*F*_(2, 21)_ =	3.57^*^	3.63^*^	3.89^*^	3.34^*^	4.67^*^	4.25^*^	3.82^*^
IV	*F*_(2, 21)_ =	12.69^***^	10.03^***^	4.26^*^	4.27^*^	6.36^**^	4.35^*^	5.45^*^
V	*F*_(2, 21)_ =	8.41^***^	6.81^**^	9.52^**^	11.48^***^	21.29^***^	4.38^*^	5.19^*^

* p < 0.05;

** p < 0.01;

*** p < 0.001.

**Figure 4:**
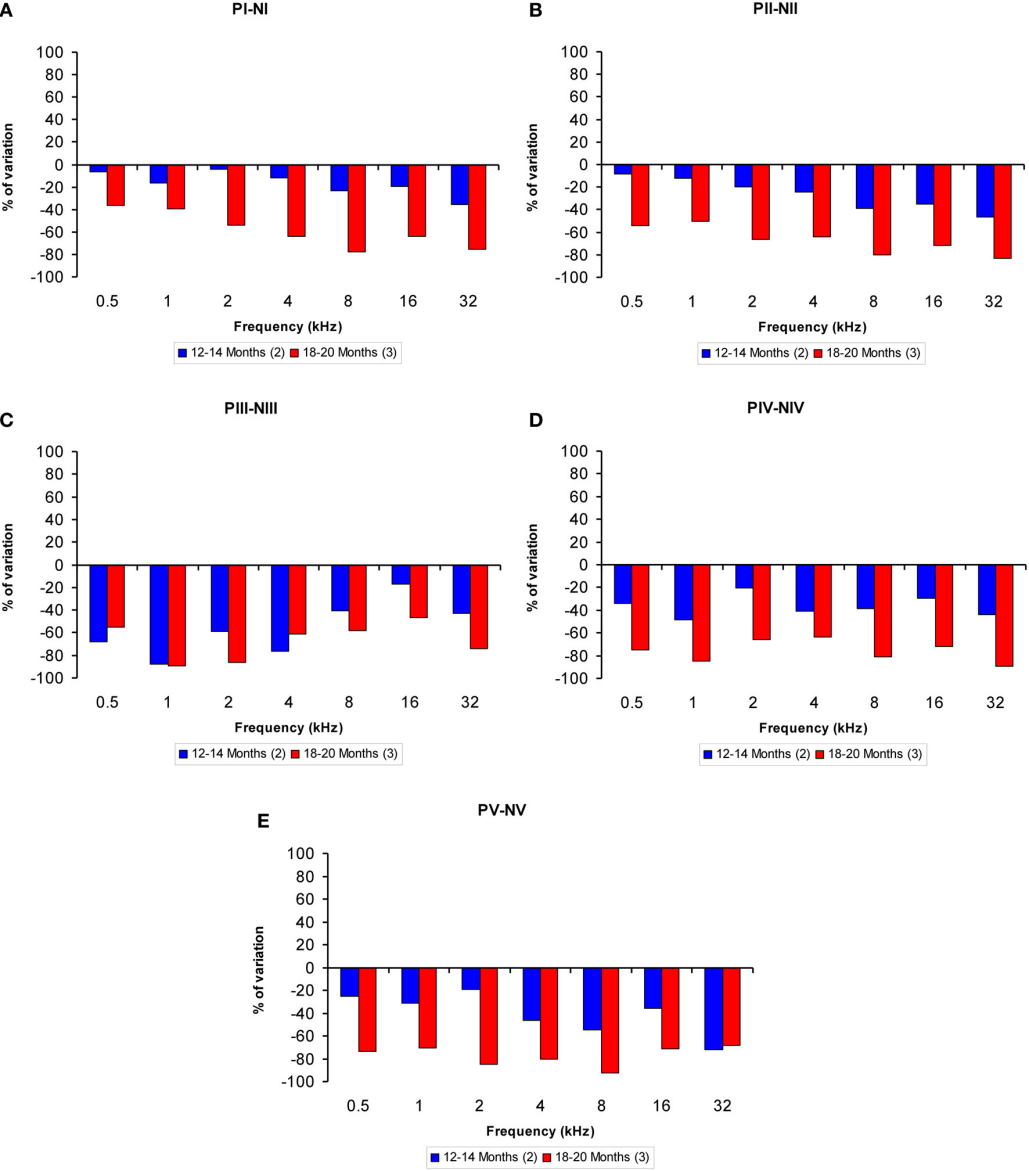
**Bar graphs illustrating the percentage of variation in the wave amplitudes in older rats compared to 6- to 8-month-old rats**. At all frequencies evaluated, compared to the 6- to 8-month-old group, the percent reduction in the wave amplitude grew as the age of the animals increased **(A–E)**.

The proportions of the variation in the wave amplitudes in the older groups compared to the 6-to 8-month-old group were calculated for all frequencies. In general, compared to the 6- to 8-month-old rats, the amplitudes of waves I, II, IV, and V were smaller in both the 12- to 14-month-old and the 18- to 20-month-old rats at higher frequencies, whereas the amplitudes of wave III were smaller at lower frequencies (Figures 4A–E). As mentioned above, this phenomenon was more apparent in the 18- to 20-month-old rats. Accordingly, for wave I, the variation ranged from −4.46 to −35.65% for the 12- to 14-month-old rats and from −36.35 to −77.61% for the 18- to 20-month-old rats (Figure 4A). For wave II, the variation ranged from −8.63 to −46.44% for the 12- to 14-month-old rats and from −54.16 to −83.40% for the 18- to 20-month-old rats (Figure 4B). For wave III, the variation ranged from −17.34 to −87.67% for the 12- to 14-month-old rats and from −46.49 to −89.10% for the 18- to 20-month-old rats (Figure 4C). With respect to wave IV, the variation ranged from −20.30 to −48.18% for the 12- to 14-month-old rats and from −63.32 to −89.14% for the 18- to 20-month-old rats (Figure 4D). Finally, for wave V, the variation ranged from −19.08 to −72.16% for the 12- to 14-month-old rats and from −68.08 to −92.32% for the 18- to 20-month-old rats (Figure 4E).

### Wave latencies

**Figure 5:**
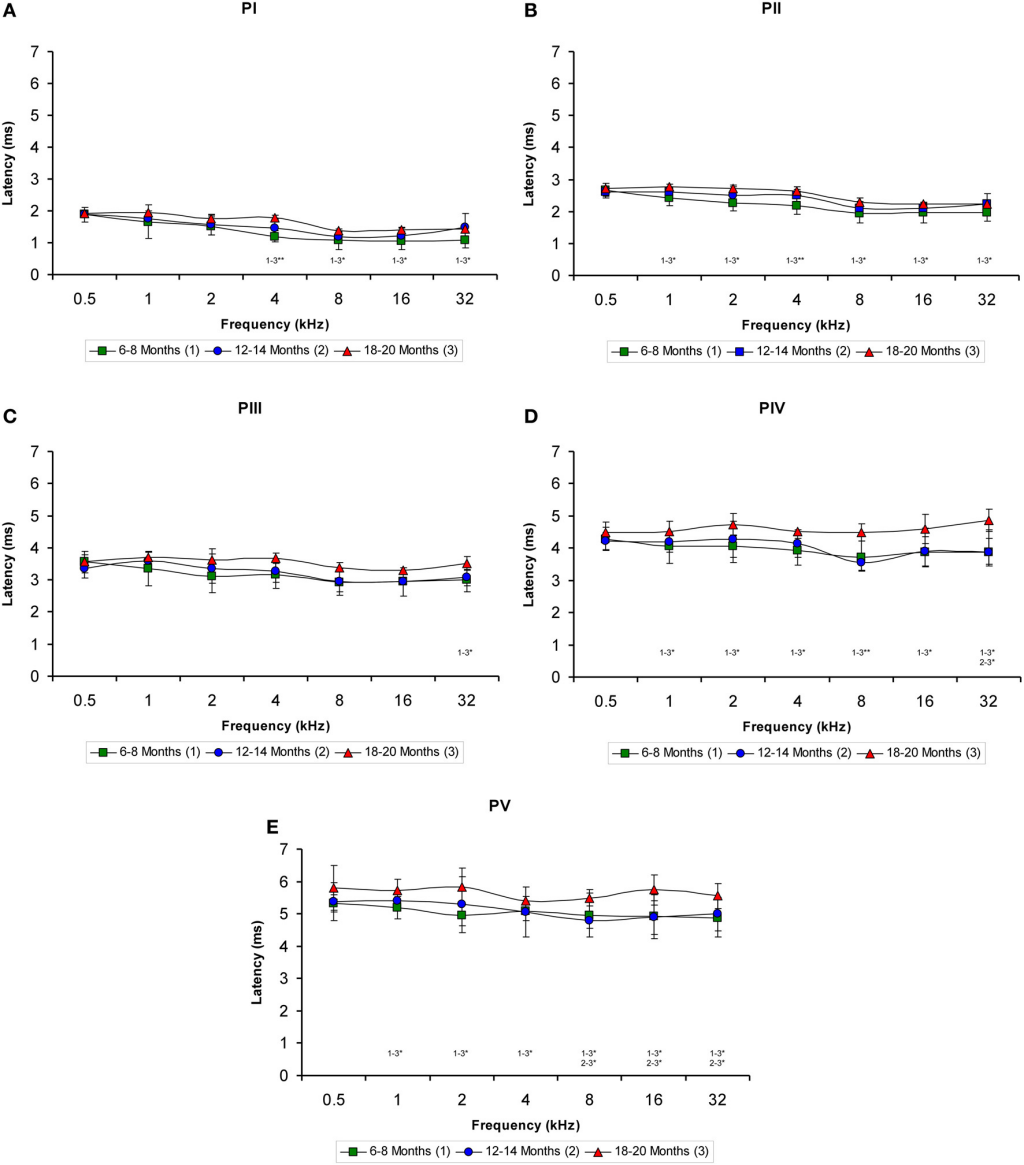
**Line graphs illustrating the positive wave latency (ms) plotted as a function of the frequency**. As shown, there was a significant effect of age on the positive wave latencies at the middle and high frequencies but not at the lower frequencies **(A–E)**. This effect was more apparent for waves IV **(D)** and V **(E)**. As indicated in the Methods section, 0.5 ms of acoustic transit time between the speaker's diaphragm and the rat's tympanic membrane was added to the latencies. Stimulus intensity = 80 dB SPL. ^*^p < 0.05; ^**^p < 0.01.

Regarding the positive wave latencies (which were measured from the stimulus onset to the peak of each wave; see Methods), although there was a significant effect of age on the wave latencies, the effect was not present at 0.5 kHz and was more apparent for waves IV and V (Figure 5). Accordingly, for wave I, significant differences were detected at 4, 8, 16, and 32 kHz (Table 4). At those frequencies, the latencies in the 18- to 20-month-old rats were longer than the latencies measured in the 6- to 8-month-old rats (Figure 5A). For wave II, significant differences were observed all frequencies except for 0.5 kHz (Table 4). The latencies of wave II were longer in the 18- to 20-month-old rats than in the 6- to 8-month-old rats (Figure 5B). Regarding wave III, there was a significant difference only at 32 kHz (Table 4), in which the latency in the 18- to 20-month-old rats was longer than the latency in the 6- to 8-month-old rats (Figure 5C). ANOVA analysis of the wave IV latencies also revealed significant differences all frequencies except for 0.5 kHz (Table 4). The wave IV latencies in the 18- to 20-month-old rats were longer than those in the 6- to 8-month-old rats at the frequencies mentioned above, and the wave IV latency in the 18–20-month-old rats was longer than the latency in the 12- to 14-month-old rats at 32 kHz (Figure 5D). Similar to waves II and IV, there were significant differences in the wave V at all frequencies except for 0.5 kHz (Table 4). The wave V latencies in the 18- to 20-month-old rats were longer than that in the 6- to 8-month-old rats and longer than the latencies in the 12- to 14-month-old rats at 8, 16, and 32 kHz (Figure 5E).

**Table 4:** **ANOVA analysis of the interaction between age of the rats and ABR wave latencies**.

		**Frequencies (kHz)**						
**Waves**		**0.5**	**1**	**2**	**4**	**8**	**16**	**32**
**POSITIVE WAVE LATENCIES**								
I	*F*_(2, 21)_ =	0.74^*NS*^	0.15^*NS*^	0.34^*NS*^	18.83^***^	4.57^*^	4.18^*^	5.98^*^
II	*F*_(2, 21)_ =	0.54^*NS*^	4.35^*^	5.58^*^	7.86^**^	4.36^*^	4.14^*^	5.01^*^
III	*F*_(2, 21)_ =	0.14^*NS*^	0.30^*NS*^	0.44^*NS*^	2.69^*NS*^	2.47^*NS*^	2.68^*NS*^	3.86^*^
IV	*F*_(2, 21)_ =	1.68^*NS*^	4.47^*^	3.82^*^	4.49^*^	5.84^*^	4.51^*^	5.09^*^
V	*F*_(2, 21)_ =	1.69^*NS*^	4.28^*^	4.40^*^	4.63^*^	4.08^*^	4.66^*^	4.88^*^
**NEGATIVE WAVE LATENCIES**								
I	*F*_(2, 21)_ =	1.54^*NS*^	3.33^*NS*^	2.82^*NS*^	7.47^**^	4.51^*^	4.50^*^	3.83^*^
II	*F*_(2, 21)_ =	0.76^*NS*^	2.89^*NS*^	4.40^*^	4.63^*^	4.08^*^	4.66^*^	4.88^*^
III	*F*_(2, 21)_ =	1.54^*NS*^	2.48^*NS*^	0.81^*NS*^	3.02^*NS*^	2.45^*NS*^	2.51^*NS*^	6.60^**^
IV	*F*_(2, 21)_ =	1.38^*NS*^	2.53^*NS*^	3.38^*NS*^	1.08^*NS*^	6.21^*^	4.13^*^	4.45^*^
V	*F*_(2, 21)_ =	1.14^*NS*^	2.90^*NS*^	2.98^*NS*^	0.57^*NS*^	1.83^*NS*^	4.77^*^	4.93^*^
**INTERPEAK LATENCIES**								
PI-PII	*F*_(2, 21)_ =	0.47^*NS*^	0.43^*NS*^	2.50^*NS*^	2.08^*NS*^	1.33^*NS*^	1.28^*NS*^	1.75^*NS*^
NI-NII	*F*_(2, 21)_ =	0.02^*NS*^	0.81^*NS*^	1.46^*NS*^	0.09^*NS*^	0.52^*NS*^	0.32^*NS*^	0.25^*NS*^
PII-PIV	*F*_(2, 21)_ =	1.13^*NS*^	0.58^*NS*^	1.05^*NS*^	0.88; 2^*NS*^	6.14^*^	4.01^*^	6.78^**^
NII-NIV	*F*_(2, 21)_ =	0.93^*NS*^	0.21^*NS*^	2.23^*NS*^	0.30; 2^*NS*^	5.63^*^	4.85^*^	4.66^*^
PI-PIV	*F*_(2, 21)_ =	1.84^*NS*^	0.53^*NS*^	2.51^*NS*^	0.07; 2^*NS*^	4.36^*^	4.68^*^	6.63^**^
NI-NIV	*F*_(2, 21)_ =	0.74^*NS*^	0.03^*NS*^	2.27^*NS*^	0.41; 2^*NS*^	4.86^*^	4.71^*^	4.62^*^

* p < 0.05;

** p < 0.01;

*** p < 0.001;

NS, No significance.

**Figure 6:**
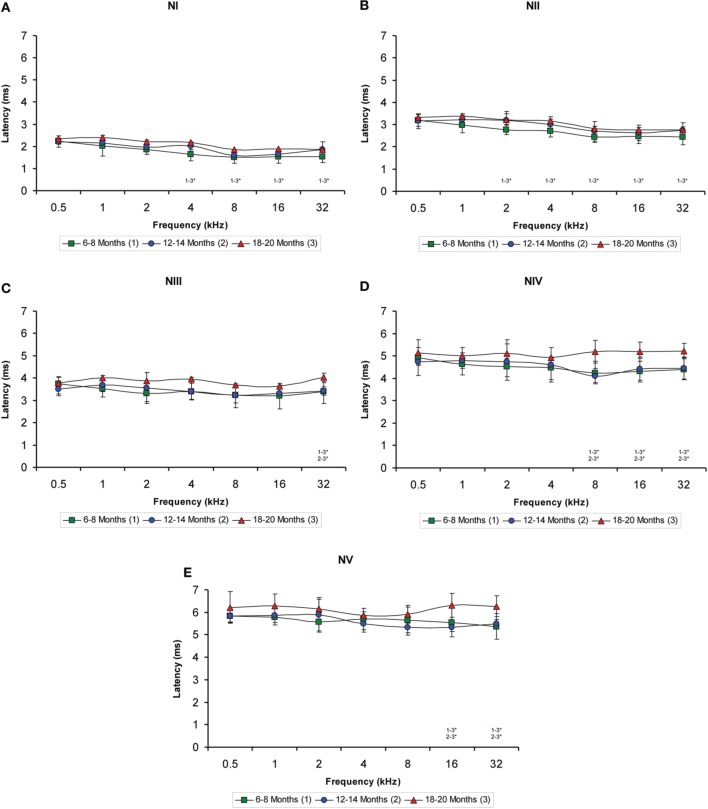
**Line graphs illustrating the negative wave latency (ms) plotted as a function of the frequency**. Similar to the positive latencies, a significant effect of age on the negative latencies of all waves was detected predominantly in the high and middle frequencies but not in the low frequencies **(A–E)**. Additionally, the effect was more apparent in waves IV **(D)** and V **(E)**. As indicated in the Methods section, 0.5 ms of acoustic transit time between the speaker's diaphragm and the rat's tympanic membrane was added to the latencies. Stimulus intensity = 80 dB SPL. ^*^p < 0.05.

When the negative wave latencies (which were measured from the stimulus onset to the negative trough of each wave; see Methods) were evaluated, a significant effect of age on the wave latencies was observed predominantly at the high and middle frequencies but not at the low frequencies (Figures 6A–E). Differences in the latencies of wave I were detected at 4, 8, 16, and 32 kHz (Table 4), in which the negative wave I latencies in the 18- to 20-month-old rats were longer than those in the 6- to 8-month-old rats (Figure 6A). There were also significant differences in the latencies of wave II at 2, 4, 8, 16, and 32 kHz (Table 4). The Scheffé *post-hoc* test revealed that the negative wave II latencies in the 18- to 20-month-old rats were also longer that the negative wave II latencies in the 6- to 8-month-old rats (Figure 6B). For wave III, significant differences were detected only at 32 kHz (Table 4), with longer wave III latencies in the 18- to 20-month-old rats compared to both the 6- to 8-month-old rats and the 12- to 14-month-old rats (Figure 6C). In the negative latencies of wave IV, significant differences were detected at 8, 16, and 32 kHz (Table 4), in which the wave IV latencies of the 18- to 20-month-old rats were longer than the wave IV latencies in the 6- to 8-month-old rats and the 12- to 14-month-old rats (Figure 6D). Finally, significant differences were detected in the negative latencies of wave V at 16 kHz and 32 kHz (Table 4), in which the wave V latencies in the 18- to 20-month-old rats were longer than those in both the 6- to 8-month-old rats and the 12- to 14-month-old rats (Figure 6E).

### Interpeak latencies

**Figure 7:**
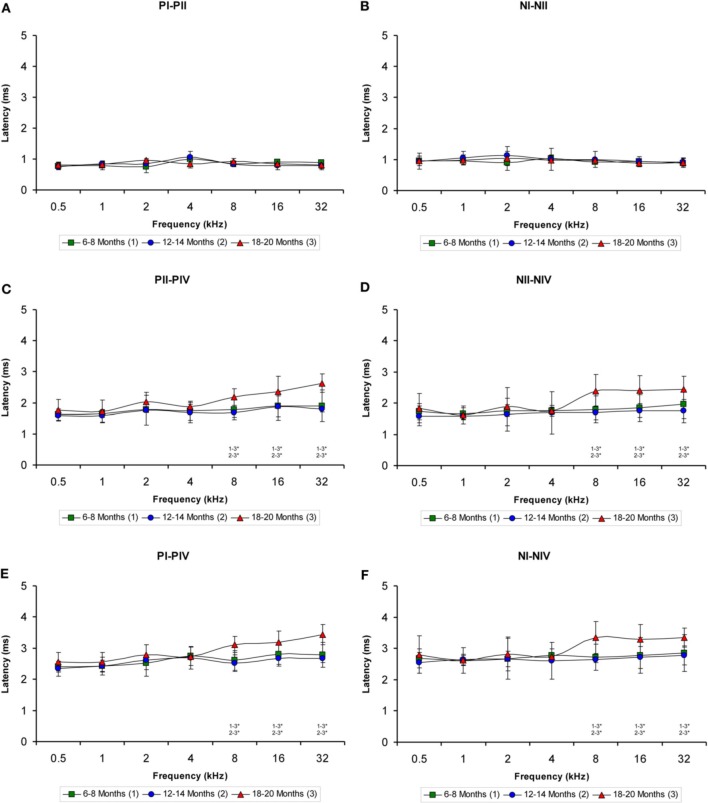
**Line graphs showing the interpeak latency (ms) plotted as a function of the frequency**. Evaluation of the interpeak latencies revealed significant differences at higher frequencies for both the positive and negative interpeak latencies between waves II and IV **(C–D)** and between waves I and IV **(E–F)**, but not between waves I and II **(A–B)** among the three age groups. Stimulus intensity = 80 dB SPL. ^*^p < 0.05.

The evaluation of the interpeak latencies via ANOVA revealed that there were no significant differences between the animal groups with respect to either the positive or the negative interpeak latencies between waves I or II (Table 4; Figures 7A,B). Conversely, significant differences were detected in both the positive and negative interpeak latencies of waves II and IV between young, middle-aged and old rats at 8, 16, and 32 kHz (Table 4). Further evaluation using Scheffé *post-hoc* analysis revealed that at those frequencies, the 18- to 20-month-old rats exhibited longer positive and negative interpeak latencies between waves II and IV compared to those in both the 6- to 8-month-old rats and the 12- to 14-month-old rats (Figures 7C,D). Similarly, significant differences in the positive and negative interpeak latencies between waves I and IV were also detected at 8, 16, and 32 kHz (Table 4). At those frequencies, the positive and negative interpeak latencies in the 18- to 20-month-old rats were longer than those in both the 6- to 8-month-old rats and 12- to 14-month-old rats (Figures 7E,F).

### VGLUT1 immunostaining

**Figure 8:**
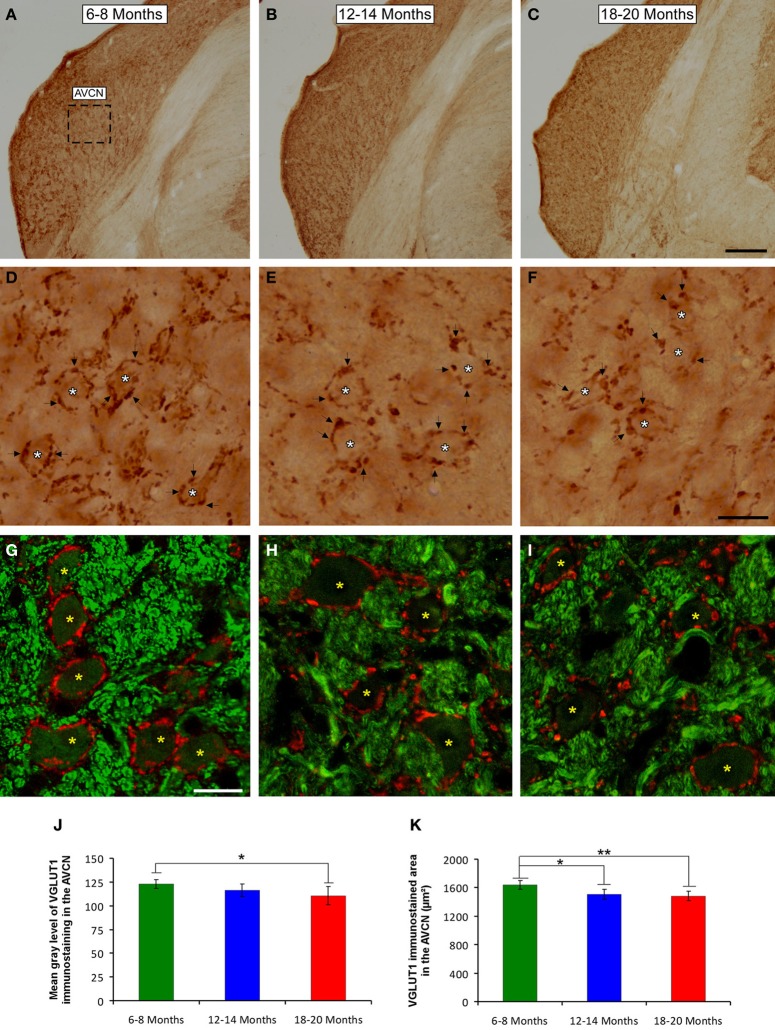
**Digitized images illustrating VGLUT1 immunoreactivity in the AVCN in 6- to 8-month-old (A,D,G), 12- to 14-month-old (B,E,H), and 18- to 20-month-old (C,F,I) Wistar rats**. In the three experimental groups, VGLUT1 immunostaining **(D–F)** appeared mainly as large profiles (arrows in **D–F**) surrounding neurons in the CN (asterisks in **D–F**), as well as small endings in the neuropil. Immunopositive profiles in 6- to 8-month-old rats **(D,G)** appeared to be more abundant than in 12- to 14-month-old **(E,H)** and 18- to 20-month-old **(F,I)** rats. Bar graphs indicate the mean gray level of VGLUT1 immunostaining **(J)** and the immunostained area **(K)** in the AVCN. The mean gray level in the 18- to 20-month-old rats **(J)** and the immunostained area in both the 12- to 14-month-old rats and the 18- to 20-month-old rats were lower compared to the 6- to 8-month-old rats **(K)**. The error bars indicate the standard errors of the mean. The square box in **A** indicates the approximate location of the high-magnification images illustrated in **D–I**. Scale bars represent 250 μm in **C**, 25 μm in **F**, and 20 μm in **G**. ^*^p < 0.05; ^**^p < 0.01.

**Figure 9:**
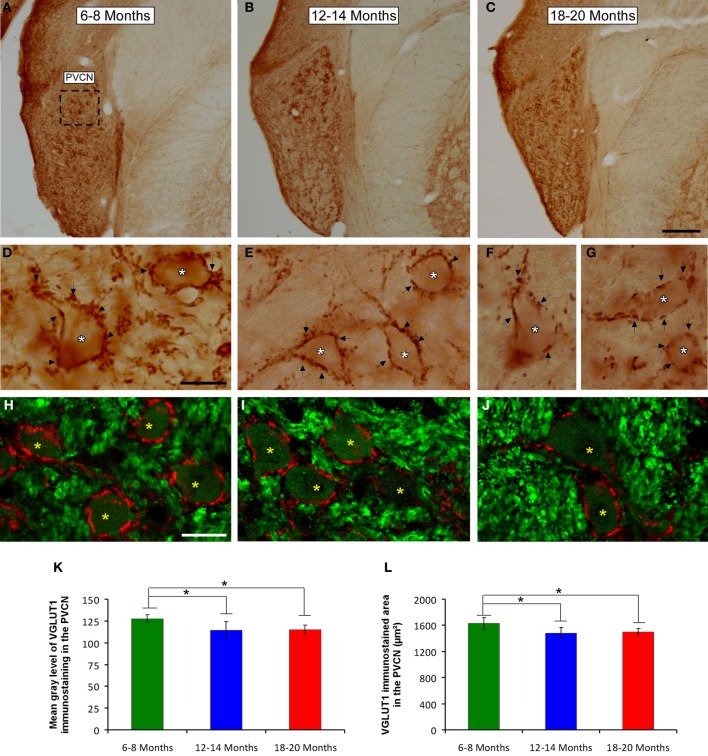
**Digitized images illustrating VGLUT1 immunostaining in the PVCN in 6- to 8-month-old (A,D,H), 12- to 14-month-old (B,E,I), and 18- to 20-month-old (C,F,G,J) rats**. Similar to the AVCN, VGLUT1 immunostaining in the PVCN consisted of perisomatic profiles (arrows and asterisks in **D–G**) and puncta throughout the neuropil. There were also noticeable differences between the groups, such that immunostaining was more profuse in the 6- to 8-month-old rats **(D,H)** in comparison to the 12- to 14-month-old **(E,I)** and 18- to 20-month-old **(F,G,J)** rats. Bar graphs indicate the mean gray level **(K)** and the area **(L)** of VGLUT1 immunostaining. The mean gray levels in both the 12- to 14-month-old and the 18- to 20-month-old rats **(K)** and the immunostained area in both the 12- to 14-month-old and in the 18- to 20-month-old rats **(L)** were lower than in the 6- to 8-month-old rats. The error bars indicate the standard errors of the mean. The square box in **A** indicates the approximate location of the high-magnification images illustrated in **D–J**. Scale bars represent 250 μm in **C**, 25 μm in **D**, and 20 μm in **H**. ^*^p < 0.05.

To determine whether the alterations in the ABR parameters detected in aged animals were related to changes in excitatory synaptic vesicles, VGLUT1 immunostaining in the ventral CN was assessed in young, middle-aged and old rats. In the three experimental groups, VGLUT1 immunostaining in the AVCN (Figures 8A–C) appeared primarily as large profiles that surrounded immunonegative somatas (arrows and asterisks in Figures 8D–I), as well as small puncta throughout the neuropil. However, although there were similarities in the pattern of distribution of VGLUT1 among the groups, there was a qualitative decrease in immunostaining as the age of the rats increased. As depicted in Figure 8, the immunopositive profiles in the samples from 6- to 8-month-old rats (Figures 8D,G) were apparently more abundant than those from both the 12- to 14-month-old rats (Figures 8E,H) and the 18- to 20-month-old rats (Figures 8F,I). Similar to the results in the AVCN, the distribution pattern of VGLUT1 in the PVCN (Figures 9A–C) also consisted of large and small immunostained profiles (arrows and asterisks in Figures 9D–J) that were more profusely distributed in the 6- to 8-month-old rats (Figures 9D,H) compared to both the 12- to 14-month-old rats (Figures 9E,I) and 18- to 20-month-old rats (Figures 9F,G,J).

To confirm these qualitative observations, densitometric analysis of VGLUT1 immunostaining was performed (see Methods). Specifically, the mean gray level and the immunostained area of the VGLUT1 immunostained profiles in both the AVCN and the PVCN were measured. In the AVCN, ANOVA revealed a significant interaction between the age of the rats and the mean gray level of VGLUT1 immunostaining [*F*_(2, 58)_ = 3.30; *p* < 0.05], such that the mean gray level was lower in the 18- to 20-month-old rats than in the 6- to 8-month-old rats (Figure 8J). There was also a significant interaction between the age of the rats and the immunostained area of VGLUT1 [*F*_(2, 58)_ = 7.71; *p* < 0.01]. Scheffé *post-hoc* analysis revealed that the immunostained area in both the 18- to 20-month-old rats and the 12- to 14-month-old rats was smaller than the immunostained area in the 6- to 8-month-old rats (Figure 8K). Similarly, in the PVCN, a significant interaction between age and the mean gray level of the profiles was observed [*F*_(2, 39)_ = 4.42; *p* < 0.05], with lower mean gray levels in both the 18- to 20-month-old rats and the 12- to 14-month-old rats compared to the 6- to 8-month-old rats (Figure 9K). Furthermore, significant differences in the immunostained area of VGLUT1 were detected in the PVCN [*F*_(2, 39)_ = 3.51; *p* < 0.05], with smaller areas in both the 18- to 20-month-old rats and the 12- to 14-month-old rats compared to the 6- to 8-month-old rats (Figure 9L).

### VGAT immunostaining

**Figure 10:**
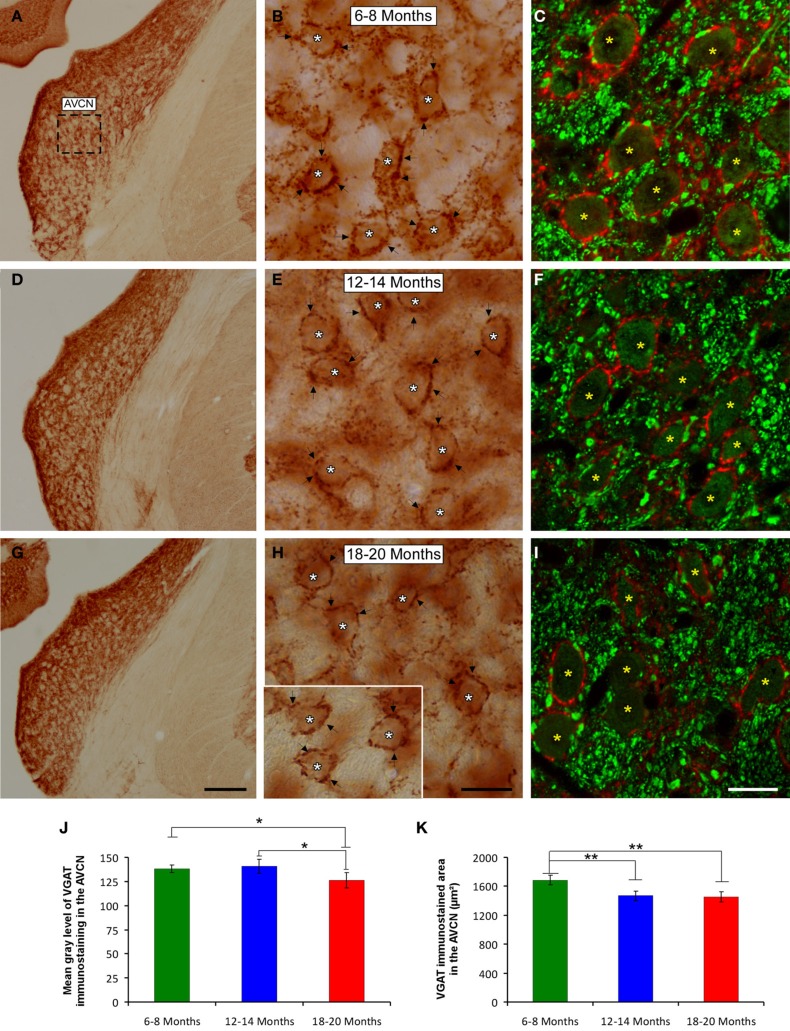
**Digitized images illustrating VGAT immunostaining in the AVCN in 6- to 8-month-old (A–C), 12- to 14-month-old (D–F), and 18- to 20-month-old (G–I) rats**. An apparent decrease in the immunostaining of large and small inhibitory synaptic endings was observed with age, with VGAT profiles (arrows and asterisks) distributed more abundantly in the 6- to 8-month-old rats **(B,C)** than in both the 12- to 14-month-old **(E,F)** and 18- to 20-month-old **(H,I)** rats. Bar graphs indicate the mean gray level **(J)** and the area **(K)** of VGAT immunostaining in the three age groups. Both the mean gray level and the immunostained area in the 18- to 20-month-old rats were significantly lower compared to both the 6- to 8-month-old rats and the 12- to 14-month-old **(J,K)** rats. The square box in **A** indicates the approximate location of the high-magnification images shown in **B,C,E,F,H,I**. Scale bars represent 250 μm in **G**, 25 μm in **H**, and 20 μm in **I**. ^*^p < 0.05; ^**^p < 0.01.

**Figure 11:**
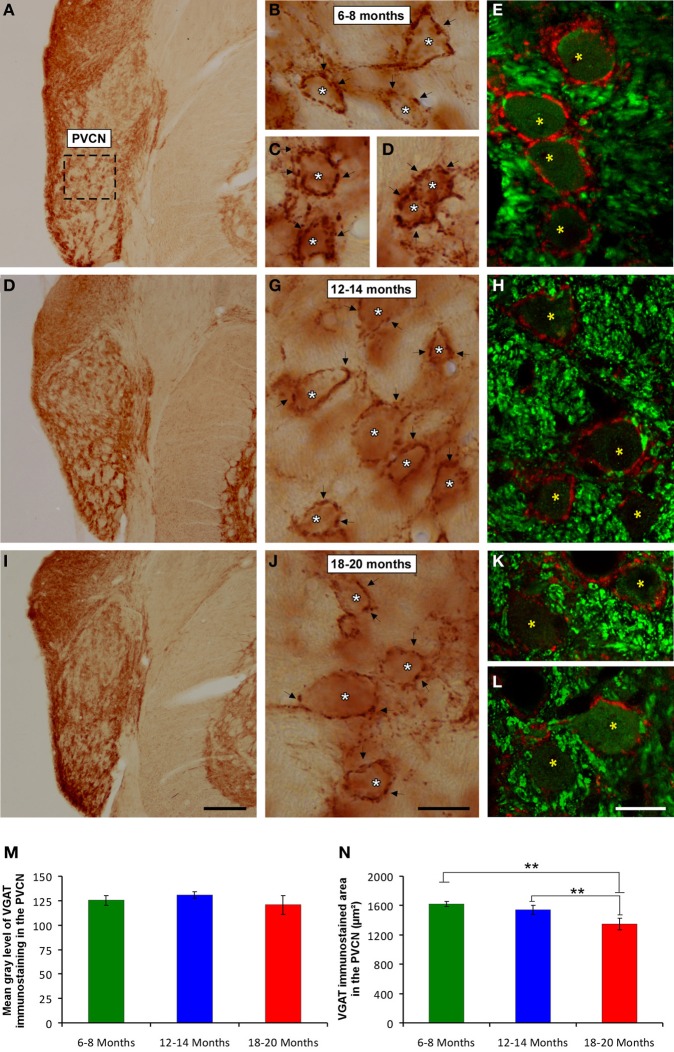
**Digitized images illustrating VGAT immunostaining in the PVCN in 6- to 8-month-old (A–E), 12- to 14-month-old (F,H) and 18- to 20-month-old (I,L) rats**. Similar to the AVCN, VGAT immunostaining was also more profuse in the 6- to 8-month-old (arrows and asterisks in **B–E**) rats than in both the 12- to 14-month-old (arrows and asterisks in **G,H**) and 18- to 20-month-old (arrows and asterisks in **J–L**) rats. Bar graphs indicate the mean gray level **(M)** and the immunostained area **(N)** of VGAT immunostaining. No differences between groups were detected in the mean gray level **(M)**, but the immunostained area **(N)** in the 18- to 20-month-old rats was significantly lower than in both the 6- to 8-month-old and 12- to 14-month-old rats. The error bars indicate the standard errors of the mean. The square box in **A** indicates the approximate location of the high-magnification images illustrated in **B–E**, **G–H**, and **J–L**. Scale bars represent 250 μm in **I**, 25 μm in **J**, and 20 μm in **L**. ^*^p < 0.05; ^**^p < 0.01.

It is feasible that decreases in excitatory synaptic vesicles in aged rats might be associated with changes in inhibitory synaptic vesicles in the CN. To address this possibility, the distribution pattern of VGAT immunostaining was investigated in the ventral CN in the three experimental groups. Regardless of the age of the rats, a survey of the coronal sections immunostained for VGAT displayed a similar distribution pattern, in which large perisomatic profiles and small synaptic endings were evenly distributed throughout the AVCN (Figure 10) and the PVCN (Figure 11). Nevertheless, an apparent age-related increase in VGAT immunostaining in the AVCN and the PVCN was detectable in the 6- to 8-month-old rats (Figures 10A–C, 11A–E) compared to both the 12- to 14-month-old rats (Figures 10D–F, 11F–H) and the 18- to 20-month-old (Figures 10G–I, 11I–L) rats.

To corroborate these findings, densitometric analysis was also performed to evaluate the mean gray level and the immunostained area of VGAT immunopositive profiles in the ventral CN (see Methods). Regarding the mean gray level in the AVCN, ANOVA revealed significant differences between the groups [*F*_(2, 46)_ = 5.87; *p* < 0.01]. Specifically, the mean gray level in the 18- to 20-month-old rats was significantly lower than in both the 6- to 8-month-old rats and in the 12- to 14-month-old rats (Figure 10J). There were significant differences between groups in the VGAT immunostained area, as well [*F*_(2, 46)_ = 13.22; *p* < 0.001], such that the VGAT immunostained area was significantly reduced in both the 18- to 20-month-old rats and the 12- to 14-month-old rats compared to the 6- to 8-month-old rats (Figure 10K). In the PVCN, although no differences were detected in the mean gray level between the age groups [*F*_(2, 41)_ = 1.72; NS] (Figure 11M), significant differences were detected in the immunostained area [*F*_(2, 41)_ = 16.24; *p* < 0.0001]. Scheffé *post-hoc* analysis revealed that the immunostained area in the 18- to 20-month-old rats was significantly smaller than the immunostained area in both the 6- to 8-month-old rats and the 12- to 14-month-old rats (Figure 11N).

## Discussion

In the present study, the evaluation of ABRs in the Wistar rat strain demonstrates that there was an increase in the auditory thresholds, a decrease in the wave amplitudes and an elongation of the wave latencies in aged animals compared to young rats. These findings were consistent with those described previously for other rat strains and for other species and are characteristic features common to animal models of ARHL (for review, see Syka, 2002, 2010; Ohlemiller, 2006; Bielefeld et al., 2010; Fetoni et al., 2011). Concomitant with the physiological alterations detected in the ABRs, there were also histological modifications, reflected by decreases in both the mean gray level and the immunostained area of VGLUT1 and VGAT in the ventral CN in older rats. The reduction in VGLUT1 immunostaining, and therefore, the strength of glutamatergic transmission (Takamori, 2006), could be partially involved in the age-related alterations in the auditory evoked responses reported in the present study. In addition, the concomitant reduction in both excitation and inhibition in central auditory nuclei might be a characteristic feature in animal models of ARLH.

It has been estimated that aged individuals (60 and over), the fastest growing age group in the world, will comprise more than 1 billion people by the end of the next decade (WHO, 2002). As the population grows older, the health problems and the sensory impairments that are associated with aging will increase. One of these sensory impairments is the presbyacusis, or ARHL, a multifactorial and complex hearing alteration that is considered to be one of the most prevalent sensory impairments among the elderly, affecting more than 30% of people over 65 years of age (WHO, 2002, 2013; Huang and Tang, 2010). This chronic condition has a profound effect on quality of life, causing social withdrawal and isolation, depression, frustration, low self-esteem and cognitive decline (WHO, 2002, 2013; Huang and Tang, 2010; Ciorba et al., 2012; Kidd III and Bao, 2012), and also has a significant economic impact, which may present an important economic burden on public health care WHO, 2002, 2013; Huang and Tang, 2010). Therefore, to improve the diagnosis, treatment and rehabilitation of patients with ARHL, is essential to understand its underlying mechanisms.

The use of animal models to study ARHL provides valuable information regarding the physiological, histological, genetic, and molecular mechanisms related to hearing and aging (Syka, 2002, 2010; Ohlemiller, 2006; Bielefeld et al., 2008, 2010; Fetoni et al., 2011). Although several species have been used for this purpose, most relevant findings have been derived from studies performed using mouse and rat models (Syka, 2002, 2010; Ohlemiller, 2006; Bielefeld et al., 2008, 2010; Fetoni et al., 2011). In the present study, the results demonstrate that Wistar rats suffer from a progressive decline in hearing, confirmed by increases in auditory thresholds as the age of the animal increases, which is a specific feature of ARHL in humans (Boettcher, 2002; Gordon-Salant, 2005; Huang and Tang, 2010; Sprinzl and Riechelmann, 2010), as well as in animal models (Syka, 2002; Bielefeld et al., 2010; Fetoni et al., 2011). These findings are consistent with those observed in the F344 rat, a widely used animal model of ARHL, which exhibits considerable increases in auditory thresholds in older animals compared to younger rats (Popelar et al., 2006; Bielefeld et al., 2008, 2010; Syka, 2010). Similar to F344 rats, hearing loss in Wistar rats begins at approximately 12–14 months of age, with a threshold shift from 24 dB in the lower frequency to 33 dB in the higher frequency. This deterioration continues such that at approximately 18–20 months of age, the auditory thresholds shift from 30 dB in the lower frequency to 46 dB in the higher frequency. The fact that the threshold shift in Wistar rats is more pronounced at higher than at lower frequencies indicates that similar to humans and other animal models, this strain exhibits more severe hearing loss at higher frequencies, which is also a hallmark of ARHL (Boettcher, 2002; Syka, 2002; Gordon-Salant, 2005; Bielefeld et al., 2010; Huang and Tang, 2010; Sprinzl and Riechelmann, 2010; Fetoni et al., 2011). It is important to note that while Wistar and F344 rats exhibit similar age-related alterations in auditory thresholds, other rat strains commonly used for the evaluation of the auditory system, such as Long-Evans rats, only exhibit small increases in auditory thresholds with age, primarily at higher frequencies and at the end of their lifespan (Popelar et al., 2006; Fetoni et al., 2011).

Age-related alterations in the hearing of Wistar rats are not only restricted to changes in auditory thresholds. Further evaluation of auditory function revealed a significant decrease in the magnitude of the auditory responses, evidenced by a decrease in the amplitude of all waveforms, which is consistent with previous findings in other animal models of ARHL, including guinea pigs (Gourévitch and Edeline, 2011), mice (Wang and Manis, 2005; Stamataki et al., 2006; Fetoni et al., 2011) and F344 rats (Backoff and Caspary, 1994; Popelar et al., 2006; Bielefeld et al., 2008), in which these animals exhibit a profound reduction in the wave amplitudes with age. In younger Wistar rats (6–8 months of age), the amplitude and morphology of the ABR waves are similar to those described previously (Chen and Chen, 1991; Overbeck and Church, 1992; Church et al., 2010, 2012a,b; Alvarado et al., 2012). However, a significant decrease in the magnitude of the responses already occurs by 12–14 month of age and becomes more pronounced in older animals (18–20 months of age) at all of the frequencies evaluated. Nevertheless, despite the decreased amplitude, the waveform morphology is preserved throughout the lifespan of Wistar rats, suggesting that the reduction in the magnitude of the responses could be due to a reduction in synaptic efficacy in the central auditory structures, as has been proposed previously in F344 rats (Popelar et al., 2006). The findings regarding the wave amplitudes in Wistar rats are in agreement with those observed in F344 rats, but differ from those reported in Long Evans rats, in which the wave amplitudes were very similar throughout their lifespan (Popelar et al., 2006).

In addition to these changes, our results also demonstrate age-related alterations in the waveform latencies in Wistar rats. As the age of the animal increases, both the positive and negative absolute latencies of all of the waves examined increase. This effect was predominant at the higher frequencies, although medium frequencies were affected as well. However, no effect of age on the wave latencies in Wistar rats was detected at all at the lower frequencies for any of the age groups evaluated. Although there is an increase in the wave latencies of all waves that comprised the ABR (positive and negative latencies), the changes were more apparent for waves IV and V, which is consistent to the results described previously in the F344 strain (Backoff and Caspary, 1994; Syka, 2010). Moreover, there is a significant prolongation of the interpeak latencies involving only waves IV and V, which is restricted to the higher frequencies in aged Wistar rats compared to younger animals. Altogether, our observations reinforce the value of Wistar rats as a model of human ARHL.

Despite the fact that F344 and Long-Evans rats exhibit similar age-related histological alterations in the cochlea, only the F344 rat strain suffers from ARHL, suggesting the additional involvement of age-related central mechanisms in the development of this sensory impairment in the F344 strain as opposed to Long-Evans rats (Popelar et al., 2006). Because Wistar rats, unlike Long-Evans rats, share other characteristic features of ARHL with F344 rats, including a reduction in the wave amplitudes and increases in the latency of waves IV and V, it is expected that Wistar rats also suffer from alterations in central auditory structures. The reduced wave amplitudes could be partially induced by a reduction in synaptic efficacy, which in turn, may be due to one factor or a combination of factors, including decreased excitatory inputs from the cochlea, impaired synaptic afferents reaching the auditory nuclei and impaired neurotransmission among the various auditory nuclei (Popelar et al., 2006). The increase in the latencies observed in the Wistar rat may reflect an increase in the conduction time between structures of the central auditory pathway (Popelar et al., 2006). Taken together, our observations support the idea that central mechanisms could also be involved in the onset and/or development of ARHL in the Wistar strain as it is in the F344 strain.

Evaluation of the cochlear nucleus of aged F344 rats demonstrated that the expression of the alpha1 and beta subunits mRNAs of the glycine receptor was significantly decreased while there was a significant increase in the mRNA expression of the alpha2 subunit. Consequently, these alterations may result in a dysfunctional receptor that may affect the binding of its ligands (Krenning et al., 1998; Syka, 2010). Based on liquid chromatography, a reduction of approximately 29% of glycine together with a concomitant reduction of approximately 24% of glutamate also has been detected in the CN of aged F344 rats compared to younger F344 rats (Banay-Schwartz et al., 1989a,b; Syka, 2010). These changes occur without an apparent loss of synapses or dendrites (Helfert et al., 2003; Syka, 2010), supporting the idea that although the structural connectivity of the CN remains relatively intact (Helfert et al., 2003; Syka, 2010), the synaptic efficacy is altered in aged F344 rats (Popelar et al., 2006) which is reflected by the aforementioned alterations in the ABR recordings (Backoff and Caspary, 1994; Popelar et al., 2006; Bielefeld et al., 2008). Our results demonstrate age-related alterations in the excitatory and inhibitory vesicular transporters in the CN of the Wistar rat. Specifically, densitometric analysis revealed a decrease in the mean gray level of VGLUT1 and VGAT immunostaining and in the immunostained area in both the AVCN and PVCN in aged animals compared to younger rats. Considering that VGLUT1 regulates the storage and release of glutamate (Kaneko and Fujiyama, 2002; Kaneko et al., 2002; Liguz-Lecznar and Skangiel-Kramska, 2007), a decrease in the levels of this vesicular transporter suggests that the strength of glutamatergic transmission in the ventral CN (Takamori, 2006), and therefore, the synaptic efficacy, could be reduced, which is consistent with the results described previously in F344 rats (Popelar et al., 2006). Such a reduction may be in part responsible for the decreased amplitude and the increased latencies of the auditory waves and, thus, the alterations in the auditory responses, detected in aged Wistar rats. Since there is not information yet about possible age-related changes in the cochlea of this strain, how peripheral modifications may contribute to the alterations in the auditory responses of Wistar rats will be an important aspect to address in future studies.

Aside from the reduction in glutamatergic function, our results also suggest a decline in inhibitory neurotransmission in the ventral CN of aged Wistar rats compared to younger animals, as indicated by decreases in the mean gray level and the immunostained area of the vesicular inhibitory (GABA and glycine) transporter (Gasnier, 2000; Buddhala et al., 2009). The fact that this concomitant reduction of excitation and inhibition also occurs in the CN of F344 rats (Banay-Schwartz et al., 1989a,b; Syka, 2010) implies that this phenomenon could be a common central alteration in animal models of ARLH. Therefore, the decreases in VGLUT1 and VGAT immunostaining in the cochlear nucleus in aged animals strengthen the hypothesis that central auditory structures are involved in the onset and/or development of ARHL in Wistar rats.

The present data clearly demonstrate that aged Wistar rats share the specific features that characterize the commonly used animal models of ARLH. Nevertheless, as animal models for ARHL, Wistar rats have an advantage over the Long-Evans strain because the latter only exhibits subtle age-related modifications in hearing, thus rendering the Long-Evans strain unsuitable for the assessment of presbyacusis (Finlayson, 2002; Campo et al., 2003; Popelar et al., 2006). Compared to the F344 strain, Wistar rats share many of the physiological alterations in the auditory system, which occur during aging (Syka, 2002; Bielefeld et al., 2010; Fetoni et al., 2011). Because Wistar rats do not suffer from any of the health problems associated with F344 rats, which exhibit a very high incidence of spontaneous tumors and degenerative disease (Popelar et al., 2006; Syka, 2010), the use of Wistar rats would avoid the risk that the evolution of hearing loss and the resulting auditory central modifications related to age could be affected by any other condition when performing long term experiments. In this regard, Wistar rats would be more suitable for the performance of experimental studies on long term therapies without the risk of interference of associated diseases that could confound the mechanism of action of the therapy examined. Also, it is important to note that Wistar rats are commonly used animal models for behavioral studies using learning paradigms involving sound. Thus, considering that age can greatly modify the hearing abilities in this strain, caution should be exercised when long-term studies are performed or when animals of different ages are used in which case it will be necessary to evaluate the auditory thresholds to ensure that the auditory function is no affected by age (Fernández-Lamo et al., 2009). In conclusion, the physiological and histological findings described in the present study support the idea that Wistar rats provide an excellent and reliable animal model to evaluate the underlying mechanisms of ARHL and also to assess new therapeutic strategies that may help to reduce the consequences of this very common sensory impairment.

## Author contributions

All authors had full access to all the data in the study and take responsibility for the integrity of the data and the accuracy of the data analysis. Study concept and design: Juan C. Alvarado, Verónica Fuentes-Santamaría, and José L. Blanco. Acquisition of data: Juan C. Alvarado, Verónica Fuentes-Santamaría, and María C. Gabaldón-Ull. Statistical analysis and interpretation of data: Juan C. Alvarado and Verónica Fuentes-Santamaría. Drafting of the manuscript: Juan C. Alvarado and Verónica Fuentes-Santamaría. Critical revision of the manuscript for important intellectual content: Juan C. Alvarado, Verónica Fuentes-Santamaría, and José M. Juiz. Obtaining funding: Juan C. Alvarado, Verónica Fuentes-Santamaría, and José M. Juiz.

### Conflict of interest statement

The authors declare that the research was conducted in the absence of any commercial or financial relationships that could be construed as a potential conflict of interest.
